# Epigenetic Regulation of Mesenchymal Stem Cells: A Focus on Osteogenic and Adipogenic Differentiation

**DOI:** 10.4061/2011/201371

**Published:** 2011-07-11

**Authors:** Chad M. Teven, Xing Liu, Ning Hu, Ni Tang, Stephanie H. Kim, Enyi Huang, Ke Yang, Mi Li, Jian-Li Gao, Hong Liu, Ryan B. Natale, Gaurav Luther, Qing Luo, Linyuan Wang, Richard Rames, Yang Bi, Jinyong Luo, Hue H. Luu, Rex C. Haydon, Russell R. Reid, Tong-Chuan He

**Affiliations:** ^1^Molecular Oncology Laboratory, Department of Surgery, The University of Chicago Medical Center, 5841 South Maryland Avenue, Chicago, IL 60637, USA; ^2^Stem Cell Biology and Therapy Laboratory, Key Laboratory for Pediatrics, The Children's Hospital of Chongqing Medical University, Chongqing 400016, China; ^3^Key Laboratory of Diagnostic Medicine, Chongqing Medical University, Chongqing 400016, China; ^4^School of Bioengineering, Chongqing University, Chongqing 400016, China; ^5^Department of Cell Biology, The Third Military Medical University, Chongqing 400038, China; ^6^Institute of Materia Medica, Zhejiang Chinese Medical University, Hangzhou 310053, China

## Abstract

Stem cells are characterized by their capability to self-renew and terminally differentiate into multiple cell types. Somatic or adult stem cells have a finite self-renewal capacity and are lineage-restricted. The use of adult stem cells for therapeutic purposes has been a topic of recent interest given the ethical considerations associated with embryonic stem (ES) cells. Mesenchymal stem cells (MSCs) are adult stem cells that can differentiate into osteogenic, adipogenic, chondrogenic, or myogenic lineages. Owing to their ease of isolation and unique characteristics, MSCs have been widely regarded as potential candidates for tissue engineering and repair. While various signaling molecules important to MSC differentiation have been identified, our complete understanding of this process is lacking. Recent investigations focused on the role of epigenetic regulation in lineage-specific differentiation of MSCs have shown that unique patterns of DNA methylation and histone modifications play an important role in the induction of MSC differentiation toward specific lineages. Nevertheless, MSC epigenetic profiles reflect a more restricted differentiation potential as compared to ES cells. Here we review the effect of epigenetic modifications on MSC multipotency and differentiation, with a focus on osteogenic and adipogenic differentiation. We also highlight clinical applications of MSC epigenetics and nuclear reprogramming.

## 1. Introduction

Two characteristics distinguish stem cells from other cell types: the ability to self-renew and to differentiate into multiple lineages. Embryonic stem (ES) cells are pluripotent cells derived from the inner cell mass of the blastocyst during early embryogenesis [1, 2]. ES cells are unique in their ability to form all cell types in the human body and self-renew indefinitely and thus have been extensively investigated in the arena of regenerative medicine since their isolation 30 years ago [1, 2]. However, ethical considerations, technical challenges, and governmental regulations have hindered their use [3]. As a result, the study of somatic or adult stem cells, which does not generate the same ethical concerns, has increased dramatically. 

Unlike ES cells, adult stem cells are characterized by a restricted differentiation potential and finite self-renewal. Adult stem cells have been localized to many tissues including mesenchymal [4], neural [5], gastrointestinal [6], hepatic [7], gonadal [8, 9], and hematopoietic [10]. Mesenchymal stem cells (MSCs) are multipotent adult stem cells that differentiate into osteoblastic, chondrogenic, myogenic, and adipogenic lineages [11–13]. MSCs are found in large numbers in the adult human, primarily in bone marrow and adipose tissue and have been widely investigated for their potential role in treating human disease. While much knowledge has been garnered regarding the characteristics and clinical applications of MSCs [14], our understanding of their behavior is still limited. Given the therapeutic potential of MSCs for a variety of conditions including bone and cartilage defects, ischemic heart disease, and cerebral ischemia, it is important that we continue to elucidate the precise mechanisms that direct MSC fate.

Though stem cell behavior is largely mediated by DNA sequence, there are multiple levels of regulation apart from this genetic blueprint including posttranscriptional, translational, posttranslational, and epigenetic regulatory processes. Epigenetic regulation is based upon heritable changes in the pattern of gene expression that occur without a change in the primary nucleotide sequence [15]. These changes remain as cells divide mitotically and meiotically and often last for multiple generations. A fundamental example of epigenetic regulation occurs as cells terminally differentiate. For example, a terminally differentiated epithelial cell shares the same DNA sequence as its ES cell precursor. However, these two cell types differ significantly in behavior and function, and some regulatory process or processes must underlie this change in phenotype. In this case, epigenetic mechanisms are largely responsible for the variable activation and repression of specific genes at specific time points during the lifespan of the cell, allowing for the terminally differentiated phenotype. Major mammalian epigenetic mechanisms include DNA methylation and histone modifications, both of which have been tightly linked to gene regulation and other cellular processes including division and survival [16, 17]. 

In recent years, epigenetic regulation has also emerged as an important modulator of stem cell differentiation [18]. Moreover, the disruption of epigenetic regulation has been associated with human disease [19]. An example of this occurs in patients with Angelman's syndrome or Prader-Willi syndrome, where epigenetic deregulation of imprinted genes at the 15q11–13 loci on the maternal or paternal allele, respectively, produces the associated phenotype [20, 21]. Epigenetic deregulation has also been implicated in many malignancies, including MSC-derived tumors [22–28]. Given its association with various disease states, epigenetic regulation has become an important focus of potential therapy. The mechanism of action of many anticancer drugs involves the alteration of DNA methylation patterns or the modification of histone proteins [29]. The therapeutic potential of epigenetic manipulation is not limited to drug therapy, however. It is also under investigation as a therapeutic modality as it relates to the process of cellular reprogramming. 

Investigation of the epigenetic regulation of cell fate determination has largely focused on ES cells. Recent studies have also elucidated epigenetic states responsible for lineage-specific differentiation of adult stem cells. While DNA methylation patterns are crucial for ES cell differentiation, histone modifications and other chromatin-based mechanisms may serve a larger role in MSC differentiation capacity [30]. Interestingly, DNA methylation profiles of MSCs suggest that, in contrast to ES cells, MSCs have a limited differentiation potential [31]. Presently, it is uncertain whether unraveling the epigenetic landscape of MSCs will lead to novel strategies to enhance their differentiation capacity. It is plausible that the heritability of gene expression in reprogrammed cells can be enhanced by the controlled manipulation of epigenetic alterations. In this paper, we summarize our current understanding of the epigenetic profile of MSCs, specifically highlighting signatures related to multipotency and differentiation into osteogenic and adipogenic lineages. We also focus on the reprogramming of MSCs and whether alterations of the MSC epigenome can enhance their therapeutic potential.

## 2. Major Epigenetic Mechanisms

Epigenetic mechanisms play a central role in the promotion of appropriate transcriptional pathways during both embryonic development and adult tissue maintenance. Regulation of gene expression at the epigenetic level occurs via modifications of chromatin architecture that alter the accessibility of genes to transcription factors and other modulators. Specifically, these modifications regulate gene expression by facilitating the opening of DNA (euchromatin) to permit transcription or the condensing of DNA (heterochromatin) to repress transcription. Loss of proper chromatin modifications during development and differentiation has been associated with embryonic lethality [32–34]. We will briefly summarize the major mechanisms underlying epigenetic regulation as they have been reviewed extensively elsewhere [35, 36].

### 2.1. DNA Methylation

Mammalian DNA methylation is unevenly dispersed over much of the genome in a pattern described as global methylation [36]. DNA methylation consists of the addition of a methyl group to position 5 of cytosine (m^5^C) at cytosine-phosphate-guanine (CpG) dinucleotides and occurs symmetrically on both DNA strands. Regions dense in CpG dinucleotides, known as CpG islands, are found near promoters of many human genes [37]. In general, promoter DNA methylation is associated with repression of the corresponding gene [38, 39]. However, this association is not always straightforward. Genes associated with methylation-free CpG islands often remain silent while genes that correspond to methylated promoters occasionally undergo transcription. This relationship may depend on the content of promoter CpG dinucleotides, where methylation of high content CpG promoters usually represses transcription, while methylation of low content CpG promoters can either activate or repress transcription [40]. Occasionally, DNA methylation may require additional epigenetic events to occur concomitantly for transcription to be affected [41].

DNA methyltransferases (DNMTs) catalyze the methylation of CpGs. Two DNMTs, DNMT3a and DNMT3b, are responsible for de novo DNA methylation during embryonic development and cell differentiation [42]. During cell division, a third DNMT, DNMT1, recognizes hemimethylated DNA and ensures methylation profile fidelity by catalyzing the methylation of its corresponding daughter strand [43]. DNA methylation is crucial for many processes including long-term gene silencing [41, 44], proper development [45–48], X chromosome inactivation [49], and genomic imprinting [50–53]. 

Though DNA methylation occurs in all cells, the unique pattern of methylation varies based on cell type [54]. Bibikova et al. [55] investigated the DNA methylation status of over 1500 CpG sites in 14 human ES cell lines and compared it to the methylation status of 38 non-ES cells lines. Using bead array and cluster analyses and methylation-specific polymerase chain reaction (PCR), the authors reported that, based on methylation profiles, human ES cells contain a unique epigenetic signature [55]. This finding may have implications on ES cell pluripotency and developmental potential. We have recently begun to uncover methylation patterns unique to MSCs as well (see below) [56, 57].

Currently, the “gold-standard” method to analyze DNA methylation patterns is bisulfite genomic sequencing [58]. This methodology consists of the bisulfite-mediated chemical conversion of unmethylated cytosine in CpG dinucleotides to uracil whereas methylated cytosines remain protected from chemical conversion [59]. PCR then substitutes uracils with thymidines and subsequent sequencing illustrates the methylation state of the original sequence. A quantitative assessment of the extent of methylation can be evaluated by bacterial cloning of the PCR products.

### 2.2. Histone Modifications

Chromatin, which is comprised of DNA and proteins, refers to the state in which DNA and these proteins are packaged within eukaryotic cell nuclei. As described above, chromatin can be packaged loosely as euchromatin, which facilitates gene transcription, or tightly as heterochromatin, which facilitates gene repression. The nucleosome is the fundamental unit of chromatin and is composed of 2 subunits of each of the four core histone proteins (H2A, H2B, H3, H4) around which 147 base pairs of DNA are wrapped. Histones are small basic proteins that are predominantly globular in nature other than their unstructured N-terminal “tails”. Subsequent to histone protein translation, N-terminal tails can be covalently modified in numerous ways to regulate gene expression [35]. The most thoroughly investigated histone modifications are acetylation and methylation.

The *histone code hypothesis* suggests that “distinct modifications, on one or more tails, act sequentially or in combination to form a “histone code” that is read by other proteins to bring about distinct downstream events” [65–67]. Histone codes can be transient or stable; if stable, these codes constitute epigenetic regulation as they imply heritability [67, 68]. Epigenetic regulation mediated by histone modification is dynamic in nature and inherently complex. For example, the methylation of histone lysine residues, catalyzed by histone methyltransferases (HMTs), can correlate with either transcriptional activation and repression [69]. Trimethylation of lysine 4 of histone H3 (H3K4me3) marks euchromatin and gene activation. In contrast, H3K27me3 and H3K9me3 signal heterochromatin and gene repression. The H3K27me3 mark is thought to be critical to the “stemness” of stem cells [70, 71], as H3K27 demethylation triggers cellular differentiation [72–74]. Further adding to histone modification complexity, the ability of HMTs to methylate H3K9 in order to silence transcription often depends on the methylation status of adjacent lysine residues on H3 [18, 75]. HMTs and histone demethylases (HDMs) work in tandem to determine the level of histone lysine methylation found within a cell [76]. 

Histone acetylation is also a widely studied histone modification. The opposing activities of histone acetyltransferases (HATs) and histone acetyl-deacetylases (HDACs) are responsible for the level of cellular histone acetylation [76]. In general, acetylation of histone lysine residues correlates with transcriptional activation whereas histone lysine deacetylation silences gene transcription. Acetylation of H3K9 (H3K9ac) and acetylation of H4K16 (H4K16ac) are common marks found on euchromatin near genes that are actively being transcribed [56]. Although histone modifications mainly act by altering chromatin architecture, specific modifications (e.g., H3K4me3 and H3K9ac) also mediate gene regulation by recruiting and tethering transcriptional modulators to chromatin [77–81]. 

Chromatin immunoprecipitation (ChIP) assays, which were originally designed to study RNA polymerase II behavior [82–85], have allowed researchers to map the positioning of histone modifications within the genome or onto individual promoters [86]. A specific histone modification can be immunoprecipitated so that DNA sequences associated with it can be identified by PCR [86]. Researchers can also indentify histone proteins that are associated with a particular region of the genome using ChIP.

Various lines of evidence suggest that chromatin within undifferentiated ES cells is generally less compact, and thus more “transcription-permissive”, compared with differentiated cells [87]. For example, pericentric heterochromatin progressively clusters as human and mouse ES cells differentiate [88, 89]. In addition, using fluorescence recovery after photobleaching (FRAP), a technique that measures the exchange rate of chromatin-associated proteins [90], Meshorer and colleagues [91] demonstrated that ES cells contain hyperdynamic chromatin proteins that loosely bind to chromatin. As ES cells begin to differentiate, these hyperdynamic proteins become immobilized on chromatin, which signal lineage commitment of these cells [91]. Indeed, the loose association of chromatin and its structural proteins may be an important marker of cellular pluripotency. Less well defined is the association of specific histone modifications to MSC cell fate. Collas et al. [56] have described the presence of bivalent histone marks (H3K4me3 and H3K27me3) on lineage-specific promoters in undifferentiated MSCs derived from adipose tissue. This finding, in addition to evidence that these same lineage-specific promoters are hypomethylated (see below), may suggest that adipogenic promoters in MSCs are preprogrammed for adipogenic stimulation [56]. 

Researchers investigating differences in histone modification patterns between epidermal stem cells and terminally differentiated cells of the epidermal lineage found that Myc-induced differentiation of adult stem cells correlates with numerous chromatin modifications [92]. Specifically, quiescent epidermal stem cells were found to contain high levels of H3K9me3 and H4K20me3 and low levels of H4 acetylation and H4K20me1 (a modification generally associated with gene activation) [92]. As Myc-treated stem cells underwent differentiation, there was a corresponding increase in H4 acetylation as well as the silencing H3K9me2 and H4K20me2 marks [92]. These data suggest that a single transcription factor has the ability to induce widespread change in chromatin state, though it remains unclear how Myc-induced differentiation of epidermal stem cells induces an increase in chromatin modifications associated with both gene activation and gene silencing. More importantly, alterations in chromatin architecture, largely mediated by epigenetic phenomena, probably underlie numerous mechanisms that facilitate cell differentiation. By elucidating avenues to manipulate such phenomena, we can potentially improve our ability to attenuate pathologies associated with tissue degeneration by directing cell fate.

## 3. Mesenchymal Stem Cells: Epigenetic Characteristics and Potency

Though MSCs have attracted significant attention for their potential to regenerate tissue, we have yet to identify a cell marker specific to MSCs. In order facilitate a more consistent approach to the study of MSC biology, the International Society of Cryotherapy has proposed that human MSCs meet the following criteria: (1) plastic adherence of cultured cells in standard culture conditions; (2) expression of CD105, CD73, and CD90 and lack of expression of CD34, CD45, CD14 or CD11b, CD79*α* or CD19, and HLA-DR surface molecules; (3) the capacity to differentiate into osteoblasts, adipocytes, and chondroblasts in vitro [93]. Populations of multipotent cells derived from adipose tissue, bone marrow, and skeletal muscle have all been found to meet these defined criteria in vitro [94–98]. Not surprisingly, these differing populations of MSCs are closely related in various capacities. For example, MSCs derived from adipose tissue (adipocyte-derived stem cells; ASCs), as well as MSCs derived from bone marrow (bone marrow MSCs; BMMSCs), express similar gene expression profiles [99–101], surface markers [94, 98], and share a similar differentiation potential [98, 102]. Sorensen et al. [103] reported that DNA methylation profiles between MSCs isolated from human adipose tissue, bone marrow, and muscle are also similar. In contrast, MSC promoter methylation profiles are distinct from other cell types, including human ES cells, multipotent ES cell-derived mesenchymal cells, and hematopoietic stem cells (HSCs) [103, 104]. 

As phenotypic, transcriptomic, functional, and now, epigenetic evidence suggests that MSCs isolated from various tissues are related, it is plausible that MSCs originate from a common origin [112]. To this end, pericytes, which have been isolated within mesodermal tissues including fat, bone, and muscle, have been found to contain several characteristic features to MSCs [113–115]. As such, authors have hypothesized that MSC populations may be traced to a pericytic origin [112, 113]. 

### 3.1. Epigenetic Profile of MSCs in Culture

In the last two decades, MSCs have been isolated from many animal [116–122] and human tissues [123–131]. Excitement regarding their use for tissue engineering purposes in part stems from the finding that MSCs navigate toward injured tissue [132] and are considered MHC II negative cells, lacking the co-stimulatory molecules CD40, CD80, and CD86 [133]. As a result, they can be allogeneically transplanted without the need for immunosuppression of the recipient. Indeed, the therapeutic potential of MSC-based treatment for a variety of conditions has already been demonstrated in humans [60–64, 134–140]. Specifically, investigators have evaluated their efficacy in the treatment of critical size bone defects, cartilage degeneration, metabolic bone disease, ischemic heart disease, and cerebral ischemia (Table 1). Nevertheless, obstacles have limited the widespread use of MSCs. In general, it has proven difficult to harvest large quantities of MSCs from many tissues, especially that of bone marrow [4]. As a result, MSCs must be expanded ex vivo after their isolation to be used for therapeutic purposes. A potential concern regarding this strategy, however, stems from the finding that MSCs display variable proliferative and differentiation capacities in culture [141]. In contrast to early-passage MSCs, late-passage MSCs have a reduced differentiation potential [142]. Moreover, MSCs may undergo malignant transformation in vitro, though this finding is controversial [143–145]. Studies addressing such concerns have documented that late-passage MSCs display normal karyotypes [146–148] and genomic stability [149], yet their behavior change in culture implies alterations of some aspect of their regulation. 

Epigenetic profiles of nonmesenchymal-derived cells occasionally display instability in culture [157–159]. To evaluate if similar phenomena occur in MSCs, Dahl and colleagues [57] examined CpG methylation patterns in human BMMSC cultures for 170 cell cycle- and cancer-related promoters. Nearly 90% of these genes maintained their methylation profile between early and late passage, indicating that MSC cultures derived from bone marrow have a stable CpG methylation status in vitro. Furthermore, the methylation profile of ASCs remains consistent up to at least 4 passages in vitro, which corresponds to 20 population doublings from a single cell [160]. Further studies are required to assess the methylation status of ASCs after longer periods in culture (i.e., after 15 passages), but it appears unlikely that alterations of promoter DNA methylation are responsible for the reduced differentiation capacity seen in late-passage MSCs [150]. 

In contrast, histone modification patterns have been implicated in variable cell behavior in culture. Under normal conditions, adipogenic and myogenic promoter regions in ASCs are associated with a bivalent combination of histone modifications. Specifically, promoter regions are enriched with H3K4me3 and H3K27me3, while lacking H3K9me3 and H3K9ac [161, 162]. As early passage ASCs differentiate, there is a corresponding rise in H3K9 acetylation and H3K27 demethylation, thereby relieving H3K4me3/H3K27me3 bivalency. In contrast, late-passage MSCs are associated with H3K27me3 maintenance and minimal H3K9 acetylation [161]. There is also global upregulation of the Polycomb repressor complex protein ExH2 (an enzyme that catalyzes the methylation of H3K27) and a global increase of H3K9 deacetylation in long-term cultured MSCs [161]. From this data, it appears that histone modification-mediated epigenetic alterations in late-passage MSCs may be responsible for a deceased ability to differentiate as cultured MSCs age. However, further studies are needed to more fully characterize this epigenetic variability on a global scale. 

### 3.2. Are MSCs Pluripotent from an Epigenetic Standpoint? 

Pluripotent cells have the ability to become cell types of all lineages in the body whereas multipotent cells differentiate into various cell types from one lineage. MSCs are often referred to as multipotent given their proclivity to form cell types within the mesodermal lineage. Recently, Jaenisch and Young [163] noted that it is unclear whether a truly pluripotent stem cell can be isolated from the adult animal. However, analyses have demonstrated that certain populations of MSCs have the ability to differentiate into cell types from all 3 germ layers [164–169]. Jiang et al. [170] localized a cell within human BMMSC cultures that, upon stimulation, could differentiate into mesenchymal, neuroectodermal, endodermal [171], and endothelial tissues [172, 173]. After injection into early blastocysts, the authors reported that this population of MSCs contributed to most or all adult cell types, thereby indicating pluripotency [170]. D'Ippolito and colleagues [174] also isolated a unique subpopulation of human bone marrow stromal cells, termed marrow-isolated adult multilineage inducible (MIAMI) cells, which could differentiate into mature cells of all 3 germ layers. Nevertheless, MSCs have not yet met more stringent criteria for pluripotency, including germline contribution or tetraploid complementation [163]. 

Presently, it is generally accepted that MSCs are confined to the mesodermal lineage, but under certain conditions can differentiate into most or all tissues. This notion of lineage-restriction has been supported by epigenetic studies from the laboratory of Collas et al. [103, 112, 160, 175]. MSC lineage-specific promoters are largely hypomethylated in MSCs. In contrast, the endothelial promoter for* CD31 *is fully methylated in ASCs and BMMSCs, which correlates with a lack of CD31 expression in MSCs. Furthermore, using methylated DNA immunoprecipitation (MeDIP), it was found that hypermethylated genes in MSCs are often associated with regulation of development, transcription, signaling, and metabolic functions. Interestingly, many promoters of genes expressed in nonmesodermal derived cells remain hypomethylated in MSCs, even though MSCs generally do not differentiate into cells that express these genes. Thus, the Collas et al. laboratory has proposed that strong methylation of lineage specification and developmental promoters may restrict MSC differentiation capacity; however, hypomethylation of lineage-specification promoters is of little predictive value in differentiation capacity [112]. This is consistent with a lineage-priming molecular model of MSC differentiation capacity, which posits that MSCs express a subset of genes corresponding to differentiation pathways to which they can commit [176]. Furthermore, in ES cells, methylation occurs on pluripotency-associated loci as cells lose pluripotency (i.e., during differentiation) [159, 177]. Taken together, epigenetic data support the notion that MSCs are better classified as multipotent than pluripotent.

## 4. Epigenetic Control of MSC Cell Fate

Questions of how the epigenetic state of a cell influences fate determination have predominately focused on ES cells. In ES cells, for example, lineage-specific promoters that are associated with terminal differentiation are often DNA methylated [178]. This presumably impedes improper or premature differentiation toward a specific lineage, thereby preserving pluripotency. Recently, studies have found that some of these same promoter regions are unmethylated in MSCs [112], indicating that the epigenetic state of ES cells changes as they differentiate into MSCs. However, whether these epigenetic alterations are the cause or result of ES cell fate decisions remains unclear. Furthermore, the mechanisms underlying MSC differentiation toward a particular cell type within the mesodermal lineage have yet to be fully elucidated. Here, we review epigenetic regulation associated with osteogenic differentiation (Table 2) and adipogenic differentiation (Table 3) of MSCs, as these pathways have been the most widely investigated.

### 4.1. Osteogenic Differentiation

Osteogenic differentiation of MSCs is a complex process that is tightly controlled by numerous signaling pathways and transcription factors [11]. Runt-related transcription factor 2 (Runx2) is considered a master regulator of osteogenic differentiation and is expressed at many stages of bone development and maturation [179–181]. Runx2 transcriptional activity is itself subject to robust regulation, as demonstrated by its association with numerous coactivators and corepressors [181–184]. It is becoming increasingly clear that epigenetic regulation is also crucial to Runx2 activity and thus osteogenic differentiation. Epigenetic regulation generally results in structural changes in chromatin that alter the binding ability of Runx2 and other transcription factors to osteogenic promoter regions. The most thoroughly studied promoter of the osteogenic lineage is the promoter for *osteocalcin *(*OC*), which contains binding sites for many factors crucial to the activation of osteoblast-specific genes including Runx2 [105, 109, 185–188]. Acetylation of histones H3 and H4, as well as a decreased level of DNA methylation, increases accessibility of the *OC* promoter to osteo-inductive transcription factors [105, 109, 185]. Furthermore, HOXA10-mediated chromatin hyperacetylation and H3K4 Trimethylation induce chromatin structural changes that facilitate Runx2-mediated activation of genes that encode OC and other osteoblastic phenotypic markers [110]. In addition, CreMM/CHD9, a recently characterized member of the CHD chromatin remodeler family [189–191], has been detected in MSCs near newly formed adult bone [192]. CReMM/CHD9 binds to promoters for both *Runx2* and *OC* during osteogenic gene expression. Though CReMM/CHD9 is thought to alter chromatin architecture via DNA-dependent ATPase activity, the exact epigenetic mechanism linking CReMM/CHD9 to osteogenic differentiation is unknown [192].

Skeletal loading and loading-induced dynamic fluid flow are also key regulators of osteogenic differentiation [193–199]. A recent investigation addressed whether these regulators act via epigenetic modifications [106]. Mechanically induced differentiation is associated with a decreased level of DNA methylation at the promoter for *osteopontin* (*OPN*; an important factor for bone remodeling) as well as increased OPN expression and osteogenic differentiation. Similarly, biologically-induced osteogenic differentiation of MSCs (using growth media supplemented with *β*-glycerolphosphate, ascorbic acid, and dexamethasone) correlates with a decrease in *OPN* promoter methylation as well as increased OPN expression [106]. 

It is not surprising that modifications of epigenetic regulation at genes crucial to osteogenic differentiation occur as MSCs become osteoblasts. However, recent evidence suggests that alterations of epigenetic regulation may occur on a more global scale as MSCs differentiate toward bone. For example, methylation at the promoter region for the mesodermal transcription factor *Brachyury*, which silences brachyury expression, is associated with osteo-induction of MSCs [107]. In addition, Hsiao and colleagues [108] reported that thyroid hormone receptor interactor-10 (*Trip10*), an adaptor protein involved in diverse cellular functions, is epigenetically modified during human BMMSC differentiation. The authors elected to investigate whether variation of *Trip10* epigenetic regulation could alter MSC differentiation patterns because of its association with the H3K27me3 mark. Interestingly, after transfection of MSCs with in vitro-methylated *Trip10* promoter DNA, MSCs underwent progressive cytosine methylation of the endogenous *Trip10* promoter, which led to reduced Trip10 expression and accelerated MSC differentiation toward osteogenic and neuronal lineages [108]. In addition to demonstrating that Trip10 expression levels are associated with osteogenic differentiation, this study illustrated how manipulation of the MSC epigenome in a manner distinct from classic nuclear reprogramming (see below) could be utilized as a therapeutic modality. However, further studies regarding the long-term effects of this type of epigenetic manipulation are necessary before it can be widely used in humans.

Support for the role of epigenetic regulation in MSC osteogenic differentiation has also come from reports of abnormal bony development. Oculo-facial-cardio-dental (OFCD) syndrome is characterized by teeth with excessively long roots and craniofacial, eye, and cardiac abnormalities [200–204]. Genetic studies have associated this X-linked dominant syndrome to mutations of the BCL-6 corepressor (BCOR) protein [204]. Under normal conditions, the repressive actions of BCOR are mediated by chromatin modifications via interactions with HDACs, HDMs, and H2A ubiquitin ligase [205–207]. MSCs have been isolated from dental and craniofacial tissues [208–210], which led Fan et al. [111] to investigate whether BCOR mutations enhance the osteo/dentinogenic potential of MSCs. Using gain- and loss-of-function assays, the authors demonstrated that the *AP-2*α** gene, a repressive target of BCOR, is largely responsible for the osteo/dentinogenic capacity of MSCs. The methylation of H3K4 and H3K36 at the *AP-2*α** promoter is associated with gene activation [211, 212]. BCOR normally catalyzes the demethylation of H3K4me3 and H3K36me2 [213, 214], but fails to do so when mutated [111]. The resultant methylation impedes the binding of the BCL-6 repressor protein to the *AP-2*α** promoter, leading to uncontrolled AP-2*α* expression. As such, a BCOR mutation that impairs its demethylating activity permits uncontrolled osteo/dentinogenic differentiation of MSCs in the OFCD syndrome [111]. 

### 4.2. Adipogenic Differentiation

The development of adipocytes during adipogenic differentiation of MSCs occurs in two phases [131, 215]. The first phase, determination, is the commitment of MSCs to the adipogenic lineage, which entails losing the ability to differentiate into another lineage. The second stage, differentiation, occurs as MSCs begin to express the phenotypic characteristics of a mature adipocyte. Similar to osteogenic differentiation, adipogenesis is a highly coordinated process that involves numerous transcription factors performing specific functions at various time points [216–218]. Just as Runx2 serves as a master regulator of osteogenic differentiation, the nuclear hormone receptor peroxisome proliferator-activated receptor-gamma (PPAR-*γ*) has a significant role in adipogenic differentiation [219, 220]. In addition, many coregulators and transcription factors central to adipogenesis have chromatin-modifying activities [221–223], supporting the role of epigenetic regulation during the differentiation of MSCs to adipocytes. 

Noer et al. [160] examined the DNA methylation profile of both adipogenic and nonadipogenic genes in human ASCs using bisulfite genomic sequencing. The promoters for four adipogenic genes—*PPAR*γ*2*, *leptin (lep)*, *fatty acid-binding protein 4 (fabp4), *and* lipoprotein lipase (lpl)*—were found to be hypomethylated in freshly harvested human ASCs [160]. Interestingly, the CpG methylation profiles between and within donors were described as mosaic (i.e., they were not uniform) [160], which is consistent with stem cells found elsewhere in the body [224, 225]. Of note, mosaic CpG methylation is believed to stem from stochastic methylating events at various CpG sites due to environmental-, health-, and age-related factors [226–229]. Noer and colleagues [160] also noted that promoter regions for housekeeping genes such as GAPDH and LMNB1 were unmethylated whereas nonadipogenic lineage-specification gene promoters (*Myogenin*, myogenic; *CD31/PECAM1 *and *CD144/CDH5*, endothelial) were hypermethylated. These findings suggest that the commitment of MSCs to the adipogenic lineage may be reflected by a particular epigenetic signature in which adipogenic gene promoters are hypomethylated while nonadipogenic promoters are methylated. In vitro analyses have correlated the demethylation of various adipogenic promoters, including that of *PPAR*γ**, with adipogenic differentiation in murine cell lines as well [151, 230, 231]. However, the pattern of promoter DNA methylation in ASCs does not always correlate with protein expression [101, 160]. This indicates that additional regulatory layers are necessary for adipogenic differentiation. 

Specific histone-mediated chromatin architecture modifications have been documented as multipotent MSCs become “preadipocytes” during determination [232]. H3K4me2, an active mark of transcription, has been identified at promoters of adipogenic genes including *adiponectin (apM1)*, *glut4*, and *lep* during determination [154]. As cells progress toward committed adipocyte precursors during differentiation, further characteristic epigenetic marks have been described. In addition to promoter DNA demethylation at *glut4 *and *lep *[152, 233, 234], these promoters also undergo H3K9 demethylation, H3 acetylation, and H3K4 Trimethylation [154, 232], all of which are epigenetic marks of gene activation. Furthermore, the downregulation of cellular HDACs during differentiation appears to facilitate adipogenic lineage commitment, while its overexpression attenuates it [155]. Interestingly, unphosphorylated retinoblastoma (Rb) protein recruits HDAC3 to promoters of *PPAR*γ** gene targets, thereby inhibiting the transcription of their associated genes and thus repressing adipogenic differentiation [156, 235]. 

MSC differentiation is required for proper tissue development and repair, but it can be detrimental when it occurs excessively. A example of this lies in the obesity epidemic currently plaguing the United States and globally [236]. As epigenetic regulation has become an increasingly recognized programming factor in the process of adipogenesis, it could follow that epigenetic deregulation has a role in the development of obesity. In fact, induced methylation alterations have been linked to obesity in mice [153, 237]. It is hopeful that further study of the epigenetic regulation of adipogenic differentiation will provide insight into potential therapies for obesity and related metabolic disorders.

## 5. Nuclear Reprogramming of Mesenchymal Stem Cells

Direct epigenetic manipulation (e.g., by transfection of methylated DNA) has not been widely used in humans because the long-term effects of such therapies are unknown. The epigenetic program of a cell can be altered in other ways, however. Several strategies have been employed to reprogram somatic cells to a pluripotent embryonic state. We will briefly summarize these strategies as they have been extensively reviewed previously [163, 238, 239]. 

Somatic cell nuclear transfer (SCNT), also referred to as nuclear transplantation (NT), is the process by which the nucleus of a somatic donor cell is introduced into an enucleated oocyte. SCNT has been used to generate cloned animals including the cloned sheep Dolly [240]. SCNT also mediates the creation of genetically matched replacement cells. As such, SCNT is of great medical interest as it has the potential to circumvent immunologic incompatibility associated with cells donated from a source other than the patient. Moreover, nuclear reprogramming of MSCs has been most widely studied in the context of SCNT. Another cellular reprogramming strategy consists of fusing somatic cells with ES cells to produce a hybrid that demonstrates ES-like features including pluripotency. However, a shortcoming of this approach is the resultant tetraploidy of reprogrammed cells. A third strategy involves the transient incubation of somatic cells with extracts of ES cells devoid of their nuclei. This method has been utilized to enhance somatic cell pluripotency in vitro without creating cells with 4 sets of chromosomes. Finally, Takahashi and Yamanaka engineered a groundbreaking nuclear reprogramming strategy with the creation of induced pluripotential stem (iPS) cells in 2006 [241]. The authors successfully reprogrammed mouse embryonic/adult fibroblasts to pluripotent ES-like stem cells (iPS cells) by introducing the transcription factors Oct4, Sox2, c-Myc, and Klf4 into differentiated cells via viral-mediated transduction [242, 243]. The ectopic expression of these reprogramming factors in infected cells initiates a sequence of epigenetic events in endogenous genes critical for the maintenance of pluripotency and lineage specification of ES cells, thereby activating the pluripotential state of iPS cells [76, 163, 241, 244]. Using a combination of similar factors, authors have also isolated iPS cells from human fibroblasts [242, 245, 246]. However, the finding that mice derived from iPS cells often develop malignancies has considerably hindered the application of this technique [247]. Interestingly, the oncogenic transcription factor c-Myc may not be necessary to reprogram cells, though it facilitates a speedy and efficient reprogramming process [246, 248, 249]. The factor or combination of factors essential to reprogramming still remains unclear, as does the specific molecular circuitry underlying pluripotency. Nevertheless, because nuclear reprogramming carries the potential to create patient-specific cells allowing for customized therapy, it is currently of great interest to many investigators.

Within the last few years, research has revealed specific epigenetic modifications that occur during the processes of differentiation and reprogramming. For example, as ES cells commit to a particular lineage, the transcription factor Oct4, which is thought to be necessary to maintain ES cell pluripotency, is rapidly silenced. Epigenetically, *Oct4* silencing correlates with a loss of gene activity marks (H3K4me3, H3K7ac, and H3K9ac), as well as an increase of gene silencing marks (H3K9me3 and DNA methylation) at the *Oct4* promoter [163, 250]. In order to generate iPS cells from somatic cells, these silencing marks must be progressively removed from the *Oct4* promoter. Furthermore, lineage-specification genes must be repressed in order to *dedifferentiate* a cell during the reprogramming process. iPS cells display chromatin modifications that prevent the transcription of genes encoding developmental regulators, thus maintaining pluripotency by repressing differentiation [163, 251, 252]. The promoters of pluripotency regulators also exhibit decreased DNA methylation patterns in iPS cells [247, 251, 252]. Taken together, these data indicate that epigenetic remodeling is an essential element to the nuclear reprogramming of somatic cells.

Although progress has been made in the field of nuclear reprogramming, there are still limitations to its therapeutic application. SCNT, for example, is an inherently inefficient process. Most clones die soon after implantation or are born with severe abnormalities due to faulty reprogramming [253, 254]. Some authors have hypothesized that, compared to terminally differentiated cells, less differentiated cell types may increase SCNT efficiency as they may be more easily reprogrammed. Faast and colleagues [255] examined if MSCs could increase the SCNT efficiency compared to terminally differentiated fibroblasts in a pig model. The use of MSCs did not increase cleavage rates compared to adult fibroblasts obtained from the same animal, but the percentage of embryos that developed to the blastocyst stage was almost doubled [255]. These findings were consistent with earlier studies that demonstrated improved SCNT efficiency using ES cells compared to differentiated somatic cells [256–260]. Jin et al. [261] also reported that compared to fetal fibroblasts, porcine MSCs have greater donor cell potential. In contrast, other investigators have noted that no significant difference exists between the number of MSCs that reach the blastocyst stage compared to fibroblasts after SCNT [262, 263]. Recently, Brero et al. [264] evaluated the efficiency of nuclear reprogramming by SCNT in MSCs and adult fibroblasts in a rabbit model by monitoring levels of histone modifications associated with transcriptionally active euchromatin (H3K4me2/3) or transcriptionally repressive facultative heterochromatin (H3K27me3). Subsequent to SCNT, H3K27me3 was found to be reprogrammed (i.e., largely undetectable) in both MSCs and adult fibroblasts, which was consistent with H3K27me3 patterns in control embryos. However, the reprogramming status of the H3K4me2/3 mark largely depended on cell type as it was inconsistent between MSCs, fibroblasts, and control embryos [264]. Based on the development of cloned embryos to the blastocyst stage as well as the level of reprogrammed histone modifications, the authors reported that MSCs were not better nucleus donors than adult fibroblasts [264]. It remains unclear why reports differ with respect to cloning efficiency using donor cells at different stages of development, but variations in methodology, technique, and species may be responsible. Further studies to address this issue will be required.

Because SCNT has proven to be an inefficient process regardless of the nuclear donor used, investigators have attempted to improve SCNT pharmacologically. The HDAC inhibitor Trichostatin A (TSA) has been shown to increase SCNT efficiency in mice [265, 266], pigs [267, 268], cattle [269, 270], and rabbits [271], though these findings are not universal [272, 273]. TSA reversibly binds to and inhibits the actions of HDACs, thereby causing acetylated histones to accumulate in cells [274]. TSA has also been shown to affect DNA methylation, DNMT expression levels, and heterochromatin remodeling [275–277]. Indeed, as epigenetic factors dynamically interact with one another, agents targeting epigenetic mechanisms have pleiotropic effects. To further evaluate the epigenetic factors modified by TSA, Martinez-Diaz and colleagues [278] assessed changes in epigenetic markers in pre- and postimplantation organism development after SCNT and TSA treatment using porcine bone marrow cells (BMCs; putative MSCs) and fetal fibroblasts. While TSA treatment increased the immunofluorescent (IF) signal of H3K14ac in embryos derived from both cell types, it did not increase the IF signal of H3K9me2. The authors also reported that TSA treatment accelerated the rate of development to the blastocyst stage for fibroblast-derived embryos but not for embryos derived from BMCs. Furthermore, embryos reconstructed from fibroblasts developed postimplantation with and without TSA treatment, whereas TSA treatment was necessary for postimplantation development in BMC-derived embryos [278]. This study partially clarified the epigenetic actions of TSA treatment during SCNT. However, it further demonstrated that the question of whether less differentiated cells (in this case BMCs) are more amenable to nuclear reprogramming than further differentiated cell types has yet to be solved.

## 6. Outlook

Stem-cell-based therapy may eventually serve as a potential remedy to many human pathologies previously thought to be incurable. MSCs in particular are promising for conditions requiring the regeneration of tissue such as bone and cartilage defects. However, before such treatments become readily available, we must further elucidate the mechanisms responsible for stem cell behavior. As we have discussed, MSCs are subject to many levels of control. Epigenetic phenomena have only recently been identified as important regulators of MSC fate. Numerous epigenetic modifications occur concomitantly during both osteogenic and adipogenic differentiation of MSCs. While much knowledge has been generated regarding the epigenetic modifications responsible for MSC differentiation, further investigation to this end will augment our ability to use MSCs therapeutically. Indeed, it is conceivable that manipulation of epigenetic signatures associated with multipotency and pluripotency, as well as modifications associated with lineage-specific differentiation, could direct patient-specific therapy. Identification of the factors necessary to reprogram mesenchymal-derived somatic cells to less differentiated states can also provide insight into the regulation of MSC fate determination.

## Figures and Tables

**Table 1:** Examples of clinical applications of mesenchymal stem cells.

Author	Year	Indication	Outcome
Bang et al. [60]	2005	Cerebral ischemia	Functional recovery after ischemic stroke improved in MSC-treated patients compared to control patients
Dill et al. [61]	2009	IHD	Intracoronary MSC administration improved LVF after STEMI
Horwitz et al. [62]	2002	Metabolic bone disease	5 of 6 OI patients showed accelerated bone growth velocity after IV infusion of allogeneic MSCs
Marcacci et al. [63]	2007	Critical size bone defect	Implantation of HA scaffolds seeded with MSCs into diaphysis defects resulted in fusion between implant and host bone
Wakitani et al. [64]	2007	Cartilage defect	Direct site transplantation of MSCs into articular cartilage defects resulted in clinical symptom improvement and defect repair

MSC: mesenchymal stem cell; IDH: ischemic heart disease; LVF: left ventricular function; STEMI: ST-segment elevated myocardial infarction; OI: osteogenesis imperfecta; IV: intravenous; HA: hydroxyapatite.

**Table 2:** Epigenetic regulation of osteogenic differentiation of mesenchymal stem cells.

Epigenetic regulation	Target	Finding	Reference
DNA methylation	*OC*	Reduced promoter DNA methylation is associated with osteogenic differentiation	Villagra et al. [105]
DNA methylation	*OPN*	Mechanically induced promoter DNA demethylation is associated with accelerated osteogenic differentiation	Arnsdorf et al. [106]
DNA methylation	*Brachyury*	Promoter DNA methylation is associated with osteogenic differentiation	Dansranjavin et al. [107]
DNA methylation	*Trip10*	Promoter DNA methylation is associated with accelerated osteogenic differentiation	Hsiao et al. [108]
Histone modification	*OC*	Acetylation of H3 and H4 is associated with OC expression and osteogenic differentiation	Shen et al. [109]
Histone modification	*HOXA10*	HOXA10-mediated chromatin acetylation and H3K4 methylation promotes transcription of osteogenic genes	Hassan et al. [110]
Histone modification	*AP-2*α**	H3K4 and H3K36 methylation is associated with AP*-*2*α* expression and subsequent osteogenic differentiation. Mutations in demethylation-related proteins (e.g., BCOR) are associated with the OFCD syndrome	Fan et al. [111]

OC: osteocalcin; OPN: osteopontin; Trip10: thyroid hormone receptor interactor-10; BCOR: BCL-6 corepressor; OFCD: oculo-facial-cardio-dental.

**Table 3:** Epigenetic regulation of adipogenic differentiation of mesenchymal stem cells.

Epigenetic regulation	Target	Finding	Reference
DNA methylation	*PPAR*γ*2*, *lep, fabp4*, *lpl *	Promoters for these 4 adipogenic genes are hypomethylated in ASCs	Noer et al. [150]
DNA methylation	*PPAR*γ**	Expression of PPAR*γ* is regulated by promoter DNA methylation. Promoter methylation corresponds to a decreased expression of PPAR*γ* and decreased adipogenic differentiation	Fujiki et al. [151]
DNA methylation	*Glut4*	Promoter DNA demethylation occurs as cells undergo adipogenic differentiation	Yokomori et al. [152]
DNA methylation	*Lep*	The *Lep *promoter region is highly methylated in preadipocytes but is unmethylated in terminally differentiated adipocytes	Melzner et al. [234]
DNA methylation	*Agouti*	Genistein-mediated DNA hypermethylation of a retrotransposon upstream of *Agouti *is associated with decreased obesity	Dolinoy et al. [153]
Histone modification	*ApM1*	H3 hyperacetylation and H3K4me3 at the *apM1 *promoter region correlate with early adipogenic differentiation. Inhibition of H3K4 methylation results in decreased apM1 expression and decreased adipogenesis	Musri et al. [154]
Histone modification	Multiple gene promoters	Downregulation of HDACs is required for adipogenic differentiation	Yoo et al. [155]
Histone modification	*PPAR*γ** gene targets	Unphosphorylated RB recruits HDAC3 to promoters of *PPAR*γ** gene targets, which decreases adipogenic differentiation. Inhibition of HDAC3 activity results in *PPAR*γ** activation, and subsequent adipogenesis	Fajas et al. [156]

PPAR*γ*: peroxisome proliferator-activated receptor-gamma; lep: leptin; fabp4: fatty acid-binding protein 4; lpl: lipoprotein lipase; ASC: adipose-derived stem cell; Glut4: glucose transporter type 4; ApM1: adiponectin; H3K4me3: Trimethylation of lysine 4 on histone 3; HDAC: histone deacetylase; RB: retinoblastoma.

## Abbreviations

ApM1: Adiponectin
ASC: Adipose-derived stem cell
BCOR: BCL-6 corepressor
BMC: Bone marrow cell
BMMSCs: Bone marrow mesenchymal stem cell
ChIP: Chromatin immunoprecipitation
CpG: Cytosine-phosphate-guanine
DNMT: DNA methyltransferase
ES: Embryonic stem
fabp4: Fatty acid-binding protein 4
FRAP: Fluorescence recovery after photobleaching
Glut4: Glucose transporter type 4
H3K9ac: Acetylation of lysine 9 on histone 3
H3K4me3: Trimethylation of lysine 4 on histone 3
H3K27me3: Trimethylation of lysine 27 on histone 3
HAT: Histone acetyltransferase
HDAC: Histone acetyl-deacetylase
HDM: Histone demethylase
HMT: Histone methyltransferase
HSC: Hematopoietic stem cell
IDH: Hschemic heart disease
IF: Immunofluorescent
iPS: Induce pluripotential stem cell
lep: Leptin
lpl: Lipoprotein lipase
LVF: Left ventricular function
MeDIP: Methylated DNA immunoprecipitation
MSC: Mesenchymal stem cell
NT: Nuclear transplantation
OC: Ostecalcin
OFCD: Oculo-facial-cardio-dental
OI: Osteogenesis imperfecta
OPN: Osteopontin
PCR: Polymerase chain reaction
PPAR*γ*: peroxisome proliferator-activated receptor-gamma
RB: retinoblastoma
Runx2: runt-related transcription factor 2
SCNT: somatic cell nuclear transfer
STEMI: ST-segment elevated myocardial infarction
Trip10: thyroid hormone receptor interactor-10
TSA: Trichostatin A.

## References

[1] Evans MJ, Kaufman MH (1981). Establishment in culture of pluripotential cells from mouse embryos. *Nature*.

[2] Martin GR (1981). Isolation of a pluripotent cell line from early mouse embryos cultured in medium conditioned by teratocarcinoma stem cells. *Proceedings of the National Academy of Sciences of the United States of America*.

[3] Frankel MS (2000). In search of stem cell policy. *Science*.

[4] Pittenger MF, Mackay AM, Beck SC (1999). Multilineage potential of adult human mesenchymal stem cells. *Science*.

[5] Gage FH (2000). Mammalian neural stem cells. *Science*.

[6] Potten CS (1998). Stem cells in gastrointestinal epithelium: numbers, characteristics and death. *Philosophical Transactions of the Royal Society B*.

[7] Alison M, Sarraf C (1998). Hepatic stem cells. *Journal of Hepatology*.

[8] Margolis J, Spradling A (1995). Identification and behavior of epithelial stem cells in the *Drosophila* ovary. *Development*.

[9] Conrad S, Renninger M, Hennenlotter J (2008). Generation of pluripotent stem cells from adult human testis. *Nature*.

[10] Weissman IL (2000). Translating stem and progenitor cell biology to the clinic: barriers and opportunities. *Science*.

[11] Deng ZL, Sharff KA, Tang N (2008). Regulation of osteogenic differentiation during skeletal development. *Frontiers in Bioscience*.

[12] Friedenstein AJ, Petrakova KV, Kurolesova AI, Frolova GP (1968). Heterotopic of bone marrow. Analysis of precursor cells for osteogenic and hematopoietic tissues. *Transplantation*.

[13] Luu HH, Song WX, Luo X (2007). Distinct roles of bone morphogenetic proteins in osteogenic differentiation of mesenchymal stem cells. *Journal of Orthopaedic Research*.

[14] Rastegar F, Shenaq D, Huang J (2010). Mesenchymal stem cells: molecular characteristics and clinical applications. *World Journal of Stem Cells*.

[15] Russo VEA, Martienssen RA, Riggs AD (1996). *Epigenetic Mechanisms of Gene Regultation*.

[16] Vincent A, Van Seuningen I (2009). Epigenetics, stem cells and epithelial cell fate. *Differentiation*.

[17] Robertson KD (2005). DNA methylation and human disease. *Nature Reviews Genetics*.

[18] Wu H, Sun YE (2006). Epigenetic regulation of stem cell differentiation. *Pediatric Research*.

[19] Egger G, Liang G, Aparicio A, Jones PA (2004). Epigenetics in human disease and prospects for epigenetic therapy. *Nature*.

[20] Nicholls RD, Saitoh S, Horsthemke B (1998). Imprinting in prader-willi and angelman syndromes. *Trends in Genetics*.

[21] Goldstone AP (2004). Prader-willi syndrome: advances in genetics, pathophysiology and treatment. *Trends in Endocrinology and Metabolism*.

[22] Kane MF, Loda M, Gaida GM (1997). Methylation of the hMLH1 promoter correlates with lack of expression of hMLH1 in sporadic colon tumors and mismatch repair-defective human tumor cell lines. *Cancer Research*.

[23] Jones PA, Baylin SB (2002). The fundamental role of epigenetic events in cancer. *Nature Reviews Genetics*.

[24] Roberts CW, Orkin SH (2004). The SWI/SNF complex—chromatin and cancer. *Nature Reviews Cancer*.

[25] Feinberg AP, Tycko B (2004). The history of cancer epigenetics. *Nature Reviews Cancer*.

[26] Soejima H, Nakagawachi T, Zhao W (2004). Silencing of imprinted *CDKN1C* gene expression is associated with loss of CpG and histone H3 lysine 9 methylation at *DMR-LIT1* in esophageal cancer. *Oncogene*.

[27] Claus R, Lubbert M (2003). Epigenetic targets in hematopoietic malignancies. *Oncogene*.

[28] Siddiqi S, Mills J, Matushansky I (2010). Epigenetic remodeling of chromatin architecture: exploring tumor differentiation therapies in mesenchymal stem cells and sarcomas. *Current Stem Cell Research & Therapy*.

[29] Ganesan A, Nolan L, Crabb SJ, Packham G (2009). Epigenetic therapy: histone acetylation, DNA methylation and anti-cancer drug discovery. *Current Cancer Drug Targets*.

[30] Collas P (2010). Programming differentiation potential in mesenchymal stem cells. *Epigenetics*.

[31] Boquest AC, Noer A, Collas P (2006). Epigenetic programming of mesenchymal stem cells from human adipose tissue. *Stem Cell Reviews*.

[32] Surani MA, Hayashi K, Hajkova P (2007). Genetic and epigenetic regulators of pluripotency. *Cell*.

[33] Tachibana M, Sugimoto K, Nozaki M (2002). G9a histone methyltransferase plays a dominant role in euchromatic histone H3 lysine 9 methylation and is essential for early embryogenesis. *Genes & Development*.

[34] Li E, Bestor TH, Jaenisch R (1992). Targeted mutation of the DNA methyltransferase gene results in embryonic lethality. *Cell*.

[35] Kouzarides T (2007). Chromatin modifications and their function. *Cell*.

[36] Bird A (2002). DNA methylation patterns and epigenetic memory. *Genes & Development*.

[37] Bird AP, Taggart MH, Nicholls RD, Higgs DR (1987). Non-methylated CpG-rich islands at the human alpha-globin locus: implications for evolution of the alpha-globin pseudogene. *The EMBO Journal*.

[38] Kass SU, Landsberger N, Wolffe AP (1997). DNA methylation directs a time-dependent repression of transcription initiation. *Current Biology*.

[39] Song F, Smith JF, Kimura MT (2005). Association of tissue-specific differentially methylated regions (TDMs) with differential gene expression. *Proceedings of the National Academy of Sciences of the United States of America*.

[40] Weber M, Hellmann I, Stadler MB (2007). Distribution, silencing potential and evolutionary impact of promoter DNA methylation in the human genome. *Nature Genetics*.

[41] Klose RJ, Bird AP (2006). Genomic DNA methylation: the mark and its mediators. *Trends in Biochemical Sciences*.

[42] Turek-Plewa J, Jagodzinski PP (2005). The role of mammalian DNA methyltransferases in the regulation of gene expression. *Cellular and Molecular Biology Letters*.

[43] Jaenisch R, Bird A (2003). Epigenetic regulation of gene expression: how the genome integrates intrinsic and environmental signals. *Nature Genetics*.

[44] Hoffman AR, Hu JF (2006). Directing DNA methylation to inhibit gene expression. *Cellular and Molecular Neurobiology*.

[45] Morgan HD, Santos F, Green K, Dean W, Reik W (2005). Epigenetic reprogramming in mammals. *Human Molecular Genetics*.

[46] Young LE, Beaujean N (2004). DNA methylation in the preimplantation embryo: the differing stories of the mouse and sheep. *Animal Reproduction Science*.

[47] Mann JR (2001). Imprinting in the germ line. *Stem Cells*.

[48] Razin A, Shemer R (1995). DNA methylation in early development. *Human Molecular Genetics*.

[49] Hellman A, Chess A (2007). Gene body-specific methylation on the active X chromosome. *Science*.

[50] Tremblay KD, Saam JR, Ingram RS, Tilghman SM, Bartolomei MS (1995). A paternal-specific methylation imprint marks the alleles of the mouse H19 gene. *Nature Genetics*.

[51] Reik W, Howlett SK, Surani MA (1990). Imprinting by DNA methylation: from transgenes to endogenous gene sequences. *Development*.

[52] Sapienza C, Peterson AC, Rossant J, Balling R (1987). Degree of methylation of transgenes is dependent on gamete of origin. *Nature*.

[53] Reik W, Collick A, Norris ML, Barton SC, Surani MA (1987). Genomic imprinting determines methylation of parental alleles in transgenic mice. *Nature*.

[54] Jaenisch R, Harbers K, Jahner D, Stewart C, Stuhlmann H (1982). DNA methylation, retroviruses, and embryogenesis. *Journal of Cellular Biochemistry*.

[55] Bibikova M, Chudin E, Wu B (2006). Human embryonic stem cells have a unique epigenetic signature. *Genome Research*.

[56] Collas P, Noer A, Sorensen AL (2008). Epigenetic basis for the differentiation potential of mesenchymal and embryonic stem cells. *Transfusion Medicine and Hemotherapy*.

[57] Dahl JA, Duggal S, Coulston N (2008). Genetic and epigenetic instability of human bone marrow mesenchymal stem cells expanded in autologous seum or fatal bovine serum. *International Journal of Developmental Biology*.

[58] Clark SJ, Statham A, Stirzaker C, Molloy PL, Frommer M (2006). DNA methylation: bisulphite modification and analysis. *Nature Protocols*.

[59] Hayatsu H, Shiraishi M, Negishi K (2008). Bisulfite modification for analysis of DNA methylation. *Current Protocols in Nucleic Acid Chemistry*.

[60] Bang OY, Lee JS, Lee PH, Lee G (2005). Autologous mesenchymal stem cell transplantation in stroke patients. *Annals of Neurology*.

[61] Dill T, Schachinger V, Rolf A (2009). Intracoronary administration of bone marrow-derived progenitor cells improves left ventricular function in patients at risk for adverse remodeling after acute ST-segment elevation myocardial infarction: results of the reinfusion of enriched progenitor cells and infarct remodeling in acute myocardial infarction study (REPAIR-AMI) cardiac magnetic resonance imaging substudy. *American Heart Journal*.

[62] Horwitz EM, Gordon PL, Koo WK (2002). Isolated allogeneic bone marrow-derived mesenchymal cells engraft and stimulate growth in children with osteogenesis imperfecta: implications for cell therapy of bone. *Proceedings of the National Academy of Sciences of the United States of America*.

[63] Marcacci M, Kon E, Moukhachev V (2007). Stem cells associated with macroporous bioceramics for long bone repair: 6- to 7-year outcome of a pilot clinical study. *Tissue Engineering*.

[64] Wakitani S, Nawata M, Tensho K, Okabe T, Machida H, Ohgushi H (2007). Repair of articular cartilage defects in the patello-femoral joint with autologous bone marrow mesenchymal cell transplantation: three case reports involving nine defects in five knees. *Journal of Tissue Engineering and Regenerative Medicine*.

[65] Strahl BD, Allis CD (2000). The language of covalent histone modifications. *Nature*.

[66] Jenuwein T, Allis CD (2001). Translating the histone code. *Science*.

[67] Lennartsson A, Ekwall K (2009). Histone modification patterns and epigenetic codes. *Biochimica et Biophysica Acta*.

[68] Turner BM (2000). Histone acetylation and an epigenetic code. *BioEssays*.

[69] Kouzarides T (2002). Histone methylation in transcriptional control. *Current Opinion in Genetics & Development*.

[70] Schwartz YB, Pirrotta V (2007). Polycomb silencing mechanisms and the management of genomic programmes. *Nature Reviews Genetics*.

[71] Simon JA, Kingston RE (2009). Mechanisms of polycomb gene silencing: knowns and unknowns. *Nature Reviews Molecular Cell Biology*.

[72] Agger K, Cloos PA, Christensen J (2007). UTX and JMJD3 are histone H3K27 demethylases involved in *HOX* gene regulation and development. *Nature*.

[73] De Santa F, Totaro MG, Prosperini E, Notarbartolo S, Testa G, Natoli G (2007). The histone H3 lysine-27 demethylase JMJD3 links inflammation to inhibition of polycomb-mediated gene silencing. *Cell*.

[74] Lan F, Bayliss PE, Rinn JL (2007). A histone H3 lysine 27 demethylase regulates animal posterior development. *Nature*.

[75] Nishioka K, Chuikov S, Sarma K (2002). Set9, a novel histone H3 methyltransferase that facilitates transcription by precluding histone tail modifications required for heterochromatin formation. *Genes & Development*.

[76] Zardo G, Cimino G, Nervi C (2008). Epigenetic plasticity of chromatin in embryonic and hematopoietic stem/progenitor cells: therapeutic potential of cell reprogramming. *Leukemia*.

[77] Zhao XD, Han X, Chew JL (2007). Whole-genome mapping of histone H3 Lys4 and 27 trimethylations reveals distinct genomic compartments in human embryonic stem cells. *Cell Stem Cell*.

[78] Bernstein BE, Mikkelsen TS, Xie X (2006). A bivalent chromatin structure marks key developmental genes in embryonic stem cells. *Cell*.

[79] Schubeler D, MacAlpine DM, Scalzo D (2004). The histone modification pattern of active genes revealed through genome-wide chromatin analysis of a higher eukaryote. *Genes & Development*.

[80] Santos-Rosa H, Schneider R, Bannister AJ (2002). Active genes are tri-methylated at K4 of histone H3. *Nature*.

[81] Struhl K (1998). Histone acetylation and transcriptional regulatory mechanisms. *Genes & Development*.

[82] Gilmour DS, Lis JT (1984). Detecting protein-DNA interactions in vivo: distribution of RNA polymerase on specific bacterial genes. *Proceedings of the National Academy of Sciences of the United States of America*.

[83] Gilmour DS, Lis JT (1985). In vivo interactions of RNA polymerase II with genes of Drosophila melanogaster. *Molecular and Cellular Biology*.

[84] Gilmour DS, Lis JT (1986). RNA polymerase II interacts with the promoter region of the noninduced hsp70 gene in Drosophila melanogaster cells. *Molecular and Cellular Biology*.

[85] Carey MF, Peterson CL, Smale ST (2009). Chromatin immunoprecipitation (ChIP). *Cold Spring Harbor Protocols*.

[86] Collas P, Dahl JA (2008). Chop it, ChIP it, check it: the current status of chromatin immunoprecipitation. *Frontiers in Bioscience*.

[87] Spivakov M, Fisher AG (2007). Epigenetic signatures of stem-cell identity. *Nature Reviews Genetics*.

[88] Wiblin AE, Cui W, Clark JA, Bickmore WA (2005). Distinctive nuclear organisation of centromeres and regions involved in pluripotency in human embryonic stem cells. *Journal of Cell Science*.

[89] Williams RR, Azuara V, Perry P (2006). Neural induction promotes large-scale chromatin reorganisation of the *Mash1* locus. *Journal of Cell Science*.

[90] Phair RD, Gorski SA, Misteli T (2004). Measurement of dynamic protein binding to chromatin *in Vivo*, using photobleaching microscopy. *Methods in Enzymology*.

[91] Meshorer E, Yellajoshula D, George E, Scambler PJ, Brown DT, Misteli T (2006). Hyperdynamic plasticity of chromatin proteins in pluripotent embryonic stem cells. *Developmental Cell*.

[92] Frye M, Fisher AG, Watt FM (2007). Epidermal stem cells are defined by global histone modifications that are altered by Myc-induced differentiation. *PLoS One*.

[93] Dominici M, Le Blanc K, Mueller I (2006). Minimal criteria for defining multipotent mesenchymal stromal cells. The international society for cellular therapy position statement. *Cytotherapy*.

[94] da Silva Meirelles L, Caplan AI, Nardi NB (2008). In search of the in Vivo identity of mesenchymal stem cells. *Stem Cells*.

[95] Peault B, Rudnicki M, Torrente Y (2007). Stem and progenitor cells in skeletal muscle development, maintenance, and therapy. *Molecular Therapy*.

[96] De Ugarte DA, Morizono K, Elbarbary A (2003). Comparison of multi-lineage cells from human adipose tissue and bone marrow. *Cells Tissues Organs*.

[97] Delorme B, Chateauvieux S, Charbord P (2006). The concept of mesenchymal stem cells. *Regenerative Medicine*.

[98] Kern S, Eichler H, Stoeve J, Kluter H, Bieback K (2006). Comparative analysis of mesenchymal stem cells from bone marrow, umbilical cord blood, or adipose tissue. *Stem Cells*.

[99] Shahdadfar A, Fronsdal K, Haug T, Reinholt FP, Brinchmann JE (2005). In vitro expansion of human mesenchymal stem cells: choice of serum is a determinant of cell proliferation, differentiation, gene expression, and transcriptome stability. *Stem Cells*.

[100] Pedemonte E, Benvenuto F, Casazza S (2007). The molecular signature of therapeutic mesenchymal stem cells exposes the architecture of the hematopoietic stem cell niche synapse. *BMC Genomics*.

[101] Boquest AC, Shahdadfar A, Fronsdal K (2005). Isolation and transcription profiling of purified uncultured human stromal stem cells: alteration of gene expression after in vitro cell culture. *Molecular Biology of the Cell*.

[102] De Ugarte DA, Alfonso Z, Zuk PA (2003). Differential expression of stem cell mobilization-associated molecules on multi-lineage cells from adipose tissue and bone marrow. *Immunology Letters*.

[103] Sorensen AL, Timoskainen S, West FD (2010). Lineage-specific promoter DNA methylation patterns segregate adult progenitor cell types. *Stem Cells and Development*.

[104] Kiel MJ, Morrison SJ (2006). Maintaining hematopoietic stem cells in the vascular niche. *Immunity*.

[105] Villagra A, Gutirrez J, Paredes R (2002). Reduced CpG methylation is associated with transcriptional activation of the bone-specific rat osteocalcin gene in osteoblasts. *Journal of Cellular Biochemistry*.

[106] Arnsdorf EJ, Tummala P, Castillo AB, Zhang F, Jacobs CR (2010). The epigenetic mechanism of mechanically induced osteogenic differentiation. *Journal of Biomechanics*.

[107] Dansranjavin T, Krehl S, Mueller T, Mueller LP, Schmoll HJ, Dammann RH (2009). The role of promoter CpG methylation in the epigenetic control of stem cell related genes during differentiation. *Cell Cycle*.

[108] Hsiao SH, Lee KD, Hsu CC (2010). DNA methylation of the Trip10 promoter accelerates mesenchymal stem cell lineage determination. *Biochemical and Biophysical Research Communications*.

[109] Shen J, Hovhannisyan H, Lian JB (2003). Transcriptional induction of the osteocalcin gene during osteoblast differentiation involves acetylation of histones H3 and H4. *Molecular Endocrinology*.

[110] Hassan MQ, Tare R, Lee SL (2007). HOXA10 controls osteoblastogenesis by directly activating bone regulatory and phenotypic genes. *Molecular and Cellular Biology*.

[111] Fan Z, Yamaza T, Lee JS (2009). BCOR regulates mesenchymal stem cell function by epigenetic mechanisms. *Nature Cell Biology*.

[112] Sorensen AL, Jacobsen BM, Reiner AH, Andersen IS, Collas P (2010). Promoter DNA methylation patterns of differentiated cells are largely programmed at the progenitor stage. *Molecular Biology of the Cell*.

[113] Crisan M, Yap S, Casteilla L (2008). A perivascular origin for mesenchymal stem cells in multiple human organs. *Cell Stem Cell*.

[114] Dellavalle A, Sampaolesi M, Tonlorenzi R (2007). Pericytes of human skeletal muscle are myogenic precursors distinct from satellite cells. *Nature Cell Biology*.

[115] Zannettino AC, Paton S, Arthur A (2008). Multipotential human adipose-derived stromal stem cells exhibit a perivascular phenotype in vitro and in vivo. *Journal of Cellular Physiology*.

[116] Igura K, Zhang X, Takahashi K, Mitsuru A, Yamaguchi S, Takahashi TA (2004). Isolation and characterization of mesenchymal progenitor cells from chorionic villi of human placenta. *Cytotherapy*.

[117] Baddoo M, Hill K, Wilkinson R (2003). Characterization of mesenchymal stem cells isolated from murine bone marrow by negative selection. *Journal of Cellular Biochemistry*.

[118] Bosnakovski D, Mizuno M, Kim G, Takagi S, Okumura M, Fujinaga T (2005). Isolation and multilineage differentiation of bovine bone marrow mesenchymal stem cells. *Cell and Tissue Research*.

[119] Devine SM, Bartholomew AM, Mahmud N (2001). Mesenchymal stem cells are capable of homing to the bone marrow of non-human primates following systemic infusion. *Experimental Hematology*.

[120] Moscoso I, Centeno A, Lopez E (2005). Differentiation “in vitro” of primary and immortalized porcine mesenchymal stem cells into cardiomyocytes for cell transplantation. *Transplantation Proceedings*.

[121] Santa Maria L, Rojas CV, Minguell JJ (2004). Signals from damaged but not undamaged skeletal muscle induce myogenic differentiation of rat bone-marrow-derived mesenchymal stem cells. *Experimental Cell Research*.

[122] Silva GV, Litovsky S, Assad JA (2005). Mesenchymal stem cells differentiate into an endothelial phenotype, enhance vascular density, and improve heart function in a canine chronic ischemia model. *Circulation*.

[123] Amoh Y, Li L, Campillo R (2005). Implanted hair follicle stem cells form Schwann cells that support repair of severed peripheral nerves. *Proceedings of the National Academy of Sciences of the United States of America*.

[124] Coles BL, Angenieux B, Inoue T (2004). Facile isolation and the characterization of human retinal stem cells. *Proceedings of the National Academy of Sciences of the United States of America*.

[125] da Silva Meirelles L, Chagastelles PC, Nardi NB (2006). Mesenchymal stem cells reside in virtually all post-natal organs and tissues. *Journal of Cell Science*.

[126] Davis AA, Temple S (1994). A self-renewing multipotential stem cell in embryonic rat cerebral cortex. *Nature*.

[127] In’t Anker PS, Scherjon SA, Kleijburg-van der Keur C (2004). Isolation of mesenchymal stem cells of fetal or maternal origin from human placenta. *Stem Cells*.

[128] Liu Z, Martin LJ (2004). Pluripotent fates and tissue regenerative potential of adult olfactory bulb neural stem and progenitor cells. *Journal of Neurotrauma*.

[129] Ringe J, Leinhase I, Stich S (2008). Human mastoid periosteum-derived stem cells: promising candidates for skeletal tissue engineering. *Journal of Tissue Engineering and Regenerative Medicine*.

[130] Sinanan AC, Hunt NP, Lewis MP (2004). Human adult craniofacial muscle-derived cells: neural-cell-adhesion-molecule (NCAM; CD56)-expressing cells appear to contain multipotential stem cells. *Biotechnology and Applied Biochemistry*.

[131] Zuk PA, Zhu M, Mizuno H (2001). Multilineage cells from human adipose tissue: implications for cell-based therapies. *Tissue Engineering*.

[132] Kollet O, Shivtiel S, Chen YQ (2003). HGF, SDF-1, and MMP-9 are involved in stress-induced human CD34^+^ stem cell recruitment to the liver. *Journal of Clinical Investigation*.

[133] Airey JA, Almeida-Porada G, Colletti EJ (2004). Human mesenchymal stem cells form purkinje fibers in fetal sheep heart. *Circulation*.

[134] Hare JM, Traverse JH, Henry TD (2009). A randomized, double-blind, placebo-controlled, dose-escalation study of Intravenous adult human mesenchymal stem cells (prochymal) after acute myocardial infarction. *Journal of the American College of Cardiology*.

[135] Horwitz EM, Prockop DJ, Fitzpatrick LA (1999). Transplantability and therapeutic effects of bone marrow-derived mesenchymal cells in children with osteogenesis imperfecta. *Nature Medicine*.

[136] Le Blanc K, Gotherstrom C, Ringden O (2005). Fetal mesenchymal stem-cell engraftment in bone after in utero transplantation in a patient with severe osteogenesis imperfecta. *Transplantation*.

[137] Quarto R, Mastrogiacomo M, Cancedda R (2001). Repair of large bone defects with the use of autologous bone marrow stromal cells. *The New England Journal of Medicine*.

[138] Kuroda R, Ishida K, Matsumoto T (2007). Treatment of a full-thickness articular cartilage defect in the femoral condyle of an athlete with autologous bone-marrow stromal cells. *Osteoarthritis and Cartilage*.

[139] Wakitani S, Mitsuoka T, Nakamura N, Toritsuka Y, Nakamura Y, Horibe S (2004). Autologous bone marrow stromal cell transplantation for repair of full-thickness articular cartilage defects in human patellae: two case reports. *Cell Transplantation*.

[140] Wakitani S, Imoto K, Yamamoto T, Saito M, Murata N, Yoneda M (2002). Human autologous culture expanded bone marrow mesenchymal cell transplantation for repair of cartilage defects in osteoarthritic knees. *Osteoarthritis and Cartilage*.

[141] Digirolamo CM, Stokes D, Colter D, Phinney DG, Class R, Prockop DJ (1999). Propagation and senescence of human marrow stromal cells in culture: a simple colony-forming assay identifies samples with the greatest potential to propagate and differentiate. *British Journal of Haematology*.

[142] Vacanti V, Kong E, Suzuki G, Sato K, Canty JM, Lee T (2005). Phenotypic changes of adult porcine mesenchymal stem cells induced by prolonged passaging in culture. *Journal of Cellular Physiology*.

[143] Rosland GV, Svendsen A, Torsvik A (2009). Long-term cultures of bone marrow-derived human mesenchymal stem cells frequently undergo spontaneous malignant transformation. *Cancer Research*.

[144] Miura M, Miura Y, Padilla-Nash HM (2006). Accumulated chromosomal instability in murine bone marrow mesenchymal stem cells leads to malignant transformation. *Stem Cells*.

[145] Tolar J, Nauta AJ, Osborn MJ (2007). Sarcoma derived from cultured mesenchymal stem cells. *Stem Cells*.

[146] Rubio D, Garcia-Castro J, Martin MC (2005). Spontaneous human adult stem cell transformation. *Cancer Research*.

[147] Miura Y, Gao Z, Miura M (2006). Mesenchymal stem cell-organized bone marrow elements: an alternative hematopoietic progenitor resource. *Stem Cells*.

[148] Zhang ZX, Guan LX, Zhang K (2007). Cytogenetic analysis of human bone marrow-derived mesenchymal stem cells passaged in vitro. *Cell Biology International*.

[149] Bernardo ME, Zaffaroni N, Novara F (2007). Human bone marrow-derived mesenchymal stem cells do not undergo transformation after long-term in vitro culture and do not exhibit telomere maintenance mechanisms. *Cancer Research*.

[150] Noer A, Boquest AC, Collas P (2007). Dynamics of adipogenic promoter DNA methylation during clonal culture of human adipose stem cells to senescence. *BMC Cell Biology*.

[151] Fujiki K, Kano F, Shiota K, Murata M (2009). Expression of the peroxisome proliferator activated receptor gamma gene is repressed by DNA methylation in visceral adipose tissue of mouse models of diabetes. *BMC Biology*.

[152] Yokomori N, Tawata M, Onaya T (1999). DNA demethylation during the differentiation of 3T3-L1 cells affects the expression of the mouse GLUT4 gene. *Diabetes*.

[153] Dolinoy DC, Weidman JR, Waterland RA, Jirtle RL (2006). Maternal genistein alters coat color and protects avy mouse offspring from obesity by modifying the fetal epigenome. *Environmental Health Perspectives*.

[154] Musri MM, Corominola H, Casamitjana R, Gomis R, Parrizas M (2006). Histone H3 lysine 4 dimethylation signals the transcriptional competence of the adiponectin promoter in preadipocytes. *Journal of Biological Chemistry*.

[155] Yoo EJ, Chung JJ, Choe SS, Kim KH,, Kim JB (2006). Down-regulation of histone deacetylases stimulates adipocyte differentiation. *Journal of Biological Chemistry*.

[156] Fajas L, Egler V, Reiter R (2002). The retinoblastoma-histone deacetylase 3 complex inhibits PPARgamma and adipocyte differentiation. *Developmental Cell*.

[157] Humpherys D, Eggan K, Akutsu H (2001). Epigenetic instability in ES cells and cloned mice. *Science*.

[158] Schumacher A, Doerfler W (2004). Influence of *in vitro* manipulation on the stability of methylation patterns in the *Snurf/Snrpn*-imprinting region in mouse embryonic stem cells. *Nucleic Acids Research*.

[159] Meissner A, Mikkelsen TS, Gu H (2008). Genome-scale DNA methylation maps of pluripotent and differentiated cells. *Nature*.

[160] Noer A, Serensen AL, Boquest AC, Collas P (2006). Stable CpG hypomethylation of adipogenic promoters in freshly isolated, cultured, and differentiated mesenchymal stem cells from adipose tissue. *Molecular Biology of the Cell*.

[161] Noer A, Lindeman LC, Collas P (2009). Histone H3 modifications associated with differentiation and long-term culture of mesenchymal adipose stem cells. *Stem Cells and Development*.

[162] Delbarre E, Jacobsen BM, Reiner AH, Sorensen AL, Kuntziger T, Collas P (2010). Chromatin environment of histone variant H3.3 revealed by quantitative imaging and genome-scale chromatin and DNA immunoprecipitation. *Molecular Biology of the Cell*.

[163] Jaenisch R, Young R (2008). Stem cells, the molecular circuitry of pluripotency and nuclear reprogramming. *Cell*.

[164] Young HE, Ceballos EM, Smith JC (1993). Pluripotent mesenchymal stem cells reside within avian connective tissue matrices. *In Vitro Cellular & Developmental Biology*.

[165] Young HE, Duplaa C, Young TM (2001). Clonogenic analysis reveals reserve stem cells in postnatal mammals: I. Pluripotent mesenchymal stem cells. *Anatomical Record*.

[166] D’Ippolito G, Diabira S, Howard GA, Roos BA, Schiller PC (2006). Low oxygen tension inhibits osteogenic differentiation and enhances stemness of human MIAMI cells. *Bone*.

[167] Young HE, Rogers JJ, Adkison LR, Lucas PA, Black AC (1998). Muscle morphogenetic protein induces myogenic gene expression in swiss-3T3 cells. *Wound Repair and Regeneration*.

[168] Rogers JJ, Young HE, Adkison LR, Lucas PA, Black AC (1995). Differentiation factors induce expression of muscle, fat, cartilage, and bone in a clone of mouse pluripotent mesenchymal stem cells. *The American Journal of Surgery*.

[169] Grigoriadis AE, Heersche JN, Aubin JE (1988). Differentiation of muscle, fat, cartilage, and bone from progenitor cells present in a bone-derived clonal cell population: effect of dexamethasone. *Journal of Cell Biology*.

[170] Jiang Y, Jahagirdar BN, Reinhardt RL (2002). Pluripotency of mesenchymal stem cells derived from adult marrow. *Nature*.

[171] Schwartz RE, Reyes M, Koodie L (2002). Multipotent adult progenitor cells from bone marrow differentiate into functional hepatocyte-like cells. *Journal of Clinical Investigation*.

[172] Reyes M, Lund T, Lenvik T, Aguiar D, Koodie L, Verfaillie CM (2001). Purification and ex vivo expansion of postnatal human marrow mesodermal progenitor cells. *Blood*.

[173] Reyes M, Dudek A, Jahagirdar B, Koodie L, Marker PH, Verfaillie CM (2002). Origin of endothelial progenitors in human postnatal bone marrow. *Journal of Clinical Investigation*.

[174] D’Ippolito G, Diabira S, Howard GA, Menei P, Roos BA, Schiller PC (2004). Marrow-isolated adult multilineage inducible (MIAMI) cells, a unique population of postnatal young and old human cells with extensive expansion and differentiation potential. *Journal of Cell Science*.

[175] Boquest AC, Noer A, Sorensen AL, Vekterud K, Collas P (2007). CpG methylation profiles of endothelial cell-specific gene promoter regions in adipose tissue stem cells suggest limited differentiation potential toward the endothelial cell lineage. *Stem Cells*.

[176] Delorme B, Ringe J, Pontikoglou C (2009). Specific lineage-priming of bone marrow mesenchymal stem cells provides the molecular framework for their plasticity. *Stem Cells*.

[177] Mohn F, Weber M, Rebhan M (2008). Lineage-specific polycomb targets and de novo DNA methylation define restriction and potential of neuronal progenitors. *Molecular Cell*.

[178] Fouse SD, Shen Y, Pellegrini M (2008). Promoter CpG methylation contributes to ES cell gene regulation in parallel with Oct4/Nanog, PcG complex, and histone H3 K4/K27 trimethylation. *Cell Stem Cell*.

[179] Karsenty G (2000). Role of Cbfa1 in osteoblast differentiation and function. *Seminars in Cell and Developmental Biology*.

[180] Ducy P, Starbuck M, Priemel M (1999). A Cbfa1-dependent genetic pathway controls bone formation beyond embryonic development. *Genes & Development*.

[181] Komori T (2006). Regulation of osteoblast differentiation by transcription factors. *Journal of Cellular Biochemistry*.

[182] Komori T (2002). Runx2, a multifunctional transcription factor in skeletal development. *Journal of Cellular Biochemistry*.

[183] Lian JB, Stein JL, Stein GS (2003). Runx2/Cbfa1 functions: diverse regulation of gene transcription by chromatin remodeling and co-regulatory protein interactions. *Connective Tissue Research*.

[184] Westendorf JJ (2006). Transcriptional co-repressors of Runx2. *Journal of Cellular Biochemistry*.

[185] Bird AP, Wolffe AP (1999). Methylation-induced repression—belts, braces, and chromatin. *Cell*.

[186] Stein GS, Lian JB, van Wijnen AJ (2004). Runx2 control of organization, assembly and activity of the regulatory machinery for skeletal gene expression. *Oncogene*.

[187] Stein GS, Lian JB, van Wijnen AJ, Stein JL (1997). The osteocalcin gene: a model for multiple parameters of skeletal-specific transcriptional control. *Molecular Biology Reports*.

[188] Montecino M, Pockwinse S, Lian J, Stein G, Stein J (1994). DNase I hypersensitive sites in promoter elements associated with basal and vitamin D dependent transcription of the bone-specific osteocalcin gene. *Biochemistry*.

[189] Shur I, Benayahu D (2005). Characterization and functional analysis of CReMM, a novel chromodomain helicase DNA-binding protein. *Journal of Molecular Biology*.

[190] Shur I, Socher R, Benayahu D (2006). In vivo association of CReMM/CHD9 with promoters in osteogenic cells. *Journal of Cellular Physiology*.

[191] Marom R, Shur I, Hager GL, Benayahu D (2006). Expression and regulation of CReMM, a chromodomain helicase-DNA-binding (CHD), in marrow stroma derived osteoprogenitors. *Journal of Cellular Physiology*.

[192] Benayahu D, Shefer G, Shur I (2009). Insights into the transcriptional and chromatin regulation of mesenchymal stem cells in musculo-skeletal tissues. *Annals of Anatomy*.

[193] Dehority W, Halloran BP, Bikle DD (1999). Bone and hormonal changes induced by skeletal unloading in the mature male rat. *American Journal of Physiology*.

[194] Lafage-Proust MH, Collet P, Dubost JM, Laroche N, Alexandre C, Vico L (1998). Space-related bone mineral redistribution and lack of bone mass recovery after reambulation in young rats. *American Journal of Physiology*.

[195] Triplett JW, O’Riley R, Tekulve K, Norvell SM, Pavalko FM (2007). Mechanical loading by fluid shear stress enhances IGF-1 receptor signaling in osteoblasts in a PKCzeta-dependent manner. *Molecular and Cellular Biomechanics*.

[196] Wronski TJ, Morey-Holton ER, Doty SB, Maese AC, Walsh CC (1987). Histomorphometric analysis of rat skeleton following spaceflight. *American Journal of Physiology*.

[197] Friedl G, Schmidt H, Rehak I, Kostner G, Schauenstein K, Windhager R (2007). Undifferentiated human mesenchymal stem cells (hMSCs) are highly sensitive to mechanical strain: transcriptionally controlled early osteo-chondrogenic response in vitro. *Osteoarthritis and Cartilage*.

[198] Kreke MR, Huckle WR, Goldstein AS (2005). Fluid flow stimulates expression of osteopontin and bone sialoprotein by bone marrow stromal cells in a temporally dependent manner. *Bone*.

[199] Li YJ, Batra NN, You L (2004). Oscillatory fluid flow affects human marrow stromal cell proliferation and differentiation. *Journal of Orthopaedic Research*.

[200] Schulze BR, Horn D, Kobelt A, Tariverdian G, Stellzig A (1999). Rare dental abnormalities seen in oculo-facio-cardio-dental (OFCD) syndrome: three new cases and review of nine patients. *American Journal of Medical Genetics*.

[201] Hedera P, Gorski JL (2003). Oculo-facio-cardio-dental syndrome: skewed X chromosome inactivation in mother and daughter suggest X-linked dominant inheritance. *American Journal of Medical Genetics*.

[202] Horn D, Chyrek M, Kleier S (2005). Novel mutations in BCOR in three patients with oculo-facio-cardio-dental syndrome, but none in Lenz microphthalmia syndrome. *European Journal of Human Genetics*.

[203] Oberoi S, Winder AE, Johnston J, Vargervik K, Slavotinek AM (2005). Case reports of oculofaciocardiodental syndrome with unusual dental findings. *American Journal of Medical Genetics*.

[204] Ng D, Thakker N, Corcoran CM (2004). Oculofaciocardiodental and Lenz microphthalmia syndromes result from distinct classes of mutations in BCOR. *Nature Genetics*.

[205] Huynh KD, Fischle W, Verdin E, Bardwell VJ (2000). BCOR, a novel corepressor involved in BCL-6 repression. *Genes & Development*.

[206] Gearhart MD, Corcoran CM, Wamstad JA, Bardwell VJ (2006). Polycomb group and SCF ubiquitin ligases are found in a novel BCOR complex that is recruited to BCL6 targets. *Molecular and Cellular Biology*.

[207] Sanchez C, Sanches I, Demmers JA, Rodriguez P, Strouboulis J, Vidal M (2007). Proteomics analysis of Ring1B/Rnf2 interactions identifies a novel complex with the Fbxl10/Jhdm1B histone demethylase and the BCL6 interacting corepressor. *Molecular & Cellular Proteomics*.

[208] Sonoyama W, Liu Y, Fang D (2006). Mesenchymal stem cell-mediated functional tooth regeneration in swine. *PLoS One*.

[209] Seo BM, Miura M, Gronthos S (2004). Investigation of multipotent postnatal stem cells from human periodontal ligament. *The Lancet*.

[210] Arthur A, Rychkov G, Shi S, Koblar SA, Gronthose S (2008). Adult human dental pulp stem cells differentiate toward functionally active neurons under appropriate environmental cues. *Stem Cells*.

[211] Shi Y, Whetstine JR (2007). Dynamic regulation of histone lysine methylation by demethylases. *Molecular Cell*.

[212] Klose RJ, Kallin EM, Zhang Y (2006). JmjC-domain-containing proteins and histone demethylation. *Nature Reviews Genetics*.

[213] Frescas D, Guardavaccaro D, Bassermann F, Koyama-Nasu R, Pagano M (2007). JHDM1B/FBXL10 is a nucleolar protein that represses transcription of ribosomal RNA genes. *Nature*.

[214] Tsukada YI, Fang J, Erdjument-Bromage H (2006). Histone demethylation by a family of JmjC domain-containing proteins. *Nature*.

[215] Otto TC, Lane MD (2005). Adipose development: from stem cell to adipocyte. *Critical Reviews in Biochemistry and Molecular Biology*.

[216] Siersbaek R, Nielsen R, Mandrup S (2010). PPARgamma in adipocyte differentiation and metabolism—novel insights from genome-wide studies. *FEBS Letters*.

[217] Farmer SR (2006). Transcriptional control of adipocyte formation. *Cell Metabolism*.

[218] MacDougald OA, Mandrup S (2002). Adipogenesis: forces that tip the scales. *Trends in Endocrinology and Metabolism*.

[219] Morrison RF, Farmer SR (1999). Insights into the transcriptional control of adipocyte differentiation. *Journal of Cellular Biochemistry*.

[220] Spiegelman BM (1998). PPAR-gamma: adipogenic regulator and thiazolidinedione receptor. *Diabetes*.

[221] Guo W, Zhang KM, Tu K (2009). Adipogenesis licensing and execution are disparately linked to cell proliferation. *Cell Research*.

[222] Martin C, Zhang Y (2005). The diverse functions of histone lysine methylation. *Nature Reviews Molecular Cell Biology*.

[223] Narlikar GJ, Fan HY, Kingston RE (2002). Cooperation between complexes that regulate chromatin structure and transcription. *Cell*.

[224] Silva AJ, Ward K, White R (1993). Mosaic methylation in clonal tissue. *Developmental Biology*.

[225] Pfeifer GP, Steigerwald SD, Hansen RS, Gartler SM, Riggs AD (1990). Polymerase chain reaction-aided genomic sequencing of an X chromosome-linked CpG island: methylation patterns suggest clonal inheritance, CpG site autonomy, and an explanation of activity state stability. *Proceedings of the National Academy of Sciences of the United States of America*.

[226] Hoffman LM, Carpenter MK (2005). Human embryonic stem cell stability. *Stem Cell Reviews*.

[227] Ushijima T, Okochi-Takada E (2005). Aberrant methylations in cancer cells: where do they come from?. *Cancer Science*.

[228] Esteller M (2005). Aberrant DNA methylation as a cancer-inducing mechanism. *Annual Review of Pharmacology and Toxicology*.

[229] Yatabe Y, Tavare S, Shibata D (2001). Investigating stem cells in human colon by using methylation patterns. *Proceedings of the National Academy of Sciences of the United States of America*.

[230] Horii T, Morita S, Kimura M, Hatada I (2009). Epigenetic regulation of adipocyte differentiation by a Rho guanine nucleotide exchange factor, WGEF. *PLoS One*.

[231] Kang MI, Kim HS, Jung YC (2007). Transitional CpG methylation between promoters and retroelements of tissue-specific genes during human mesenchymal cell differentiation. *Journal of Cellular Biochemistry*.

[232] Musri MM, Gomis R, Parrizas M (2007). Chromatin and chromatin-modifying proteins in adipogenesis. *Biochemistry and Cell Biology*.

[233] Yokomori N, Tawata M, Onaya T (2002). DNA demethylation modulates mouse leptin promoter activity during the differentiation of 3T3-L1 cells. *Diabetologia*.

[234] Melzner I, Scott V, Dorsch K (2002). Leptin gene expression in human preadipocytes is switched on by maturation-induced demethylation of distinct CpGs in its proximal promoter. *Journal of Biological Chemistry*.

[235] Feige JN, Auwerx J (2007). Transcriptional coregulators in the control of energy homeostasis. *Trends in Cell Biology*.

[236] Liu L, Li Y, Tollefsbol TO (2008). Gene-environment interactions and epigenetic basis of human diseases. *Current Issues in Molecular Biology*.

[237] Miltenberger RJ, Mynatt RL, Wilkinson JE, Woychik RP (1997). The role of the agouti gene in the yellow obese syndrome. *Journal of Nutrition*.

[238] Yamanaka S (2007). Strategies and new developments in the generation of patient-specific pluripotent stem cells. *Cell Stem Cell*.

[239] Hochedlinger K, Jaenisch R (2006). Nuclear reprogramming and pluripotency. *Nature*.

[240] Wilmut I, Schnieke AE, McWhir J, Kind AJ, Campbell KH (1997). Viable offspring derived from fetal and adult mammalian cells. *Nature*.

[241] Takahashi K, Yamanaka S (2006). Induction of pluripotent stem cells from mouse embryonic and adult fibroblast cultures by defined factors. *Cell*.

[242] Takahashi K, Tanabe K, Ohnuki M (2007). Induction of pluripotent stem cells from adult human fibroblasts by defined factors. *Cell*.

[243] Takahashi K, Okita K, Nakagawa M, Yamanaka S (2007). Induction of pluripotent stem cells from fibroblast cultures. *Nature Protocols*.

[244] Niwa H (2007). How is pluripotency determined and maintained?. *Development*.

[245] Park IH, Zhao R, West JA (2008). Reprogramming of human somatic cells to pluripotency with defined factors. *Nature*.

[246] Yu J, Vodyanik MA, Smuga-Otto K (2007). Induced pluripotent stem cell lines derived from human somatic cells. *Science*.

[247] Okita K, Ichisaka T, Yamanaka S (2007). Generation of germline-competent induced pluripotent stem cells. *Nature*.

[248] Nakagawa M, Koyanagi M, Tanabe K (2008). Generation of induced pluripotent stem cells without Myc from mouse and human fibroblasts. *Nature Biotechnology*.

[249] Wernig M, Meissner A, Cassady JP, Jaenisch R (2008). c-Myc is dispensable for direct reprogramming of mouse fibroblasts. *Cell Stem Cell*.

[250] Feldman N, Gerson A, Fang J (2006). G9a-mediated irreversible epigenetic inactivation of Oct-3/4 during early embryogenesis. *Nature Cell Biology*.

[251] Wernig M, Meissner A, Foreman R (2007). In vitro reprogramming of fibroblasts into a pluripotent ES-cell-like state. *Nature*.

[252] Maherali N, Sridharan R, Xie W (2007). Directly reprogrammed fibroblasts show global epigenetic remodeling and widespread tissue contribution. *Cell Stem Cell*.

[253] Yang X, Smith SL, Tian XC, Lewin HA, Renard JP, Wakayama T (2007). Nuclear reprogramming of cloned embryos and its implications for therapeutic cloning. *Nature Genetics*.

[254] Hochedlinger K, Jaenisch R (2003). Nuclear transplantation, embryonic stem cells, and the potential for cell therapy. *The New England Journal of Medicine*.

[255] Faast R, Harrison SJ, Beebe LF, McIlfatrick SM, Ashman RJ, Nottle MB (2006). Use of adult mesenchymal stem cells isolated from bone marrow and blood for somatic cell nuclear transfer in pigs. *Cloning and Stem Cells*.

[256] Jaenisch R, Eggan K, Humpherys D, Rideout W, Hochedlinger K (2002). Nuclear cloning, stem cells, and genomic reprogramming. *Cloning and Stem Cells*.

[257] Eggan K, Akutsu H, Loring J (2001). Hybrid vigor, fetal overgrowth, and viability of mice derived by nuclear cloning and tetraploid embryo complementation. *Proceedings of the National Academy of Sciences of the United States of America*.

[258] Eggan K, Rode A, Jentsch I (2002). Male and female mice derived from the same embryonic stem cell clone by tetraploid embryo complementation. *Nature Biotechnology*.

[259] Rideout WM, Wakayama T, Wutz A (2000). Generation of mice from wild-type and targeted ES cells by nuclear cloning. *Nature Genetics*.

[260] Wakayama T, Rodriguez I, Perry AC, Yanagimachi R, Mombaerts P (1999). Mice cloned from embryonic stem cells. *Proceedings of the National Academy of Sciences of the United States of America*.

[261] Jin HF, Kumar BM, Kim JG (2007). Enhanced development of porcine embryos cloned from bone marrow mesenchymal stem cells. *International Journal of Developmental Biology*.

[262] Bosch P, Pratt SL, Stice SL (2006). Isolation, characterization, gene modification, and nuclear reprogramming of porcine mesenchymal stem cells. *Biology of Reproduction*.

[263] Colleoni S, Donofrio G, Lagutina I, Duchi R, Galli C, Lazzari G (2005). Establishment, differentiation, electroporation, viral transduction, and nuclear transfer of bovine and porcine mesenchymal stem cells. *Cloning and Stem Cells*.

[264] Brero A, Hao R, Schieker M (2009). Reprogramming of active and repressive histone modifications following nuclear transfer with rabbit mesenchymal stem cells and adult fibroblasts. *Cloning and Stem Cells*.

[265] Kishigami S, Bui HT, Wakayama S (2007). Successful mouse cloning of an outbred strain by trichostatin a treatment after somatic nuclear transfer. *The Journal of Reproduction and Development*.

[266] Kishigami S, Mizutani E, Ohta H (2006). Significant improvement of mouse cloning technique by treatment with trichostatin a after somatic nuclear transfer. *Biochemical and Biophysical Research Communications*.

[267] Li J, Svarcova O, Villemoes K (2008). High in vitro development after somatic cell nuclear transfer and trichostatin A treatment of reconstructed porcine embryos. *Theriogenology*.

[268] Zhang Y, Li J, Villemoes K, Pedersen AM, Purup S, Vajta G (2007). An epigenetic modifier results in improved in vitro blastocyst production after somatic cell nuclear transfer. *Cloning and Stem Cells*.

[269] Iager AE, Ragina NP, Ross PJ (2008). Trichostatin A improves histone acetylation in bovine somatic cell nuclear transfer early embryos. *Cloning and Stem Cells*.

[270] Ding X, Wang Y, Zhang D, Wang Y, Guo Z, Zhang Y (2008). Increased pre-implantation development of cloned bovine embryos treated with 5-aza-2′-deoxycytidine and trichostatin A. *Theriogenology*.

[271] Shi LH, Miao YL, Ouyang YC (2008). Trichostatin A (TSA) improves the development of rabbit-rabbit intraspecies cloned embryos, but not rabbit-human interspecies cloned embryos. *Developmental Dynamics*.

[272] Meng Q, Polgar Z, Liu J, Dinnyes A (2009). Live birth of somatic cell-cloned rabbits following trichostatin A treatment and cotransfer of parthenogenetic embryos. *Cloning and Stem Cells*.

[273] Wu X, Li Y, Li GP (2008). Trichostatin A improved epigenetic modifications of transfected cells but did not improve subsequent cloned embryo development. *Animal Biotechnology*.

[274] Marks PA, Richon VM, Rifkind RA (2000). Histone deacetylase inhibitors: inducers of differentiation or apoptosis of transformed cells. *Journal of the National Cancer Institute*.

[275] Maalouf WE, Liu Z, Brochard V (2009). Trichostatin A treatment of cloned mouse embryos improves constitutive heterochromatin remodeling as well as developmental potential to term. *BMC Developmental Biology*.

[276] Li X, Kato Y, Tsuji Y, Tsunoda Y (2008). The effects of trichostatin A on mRNA expression of chromatin structure-, DNA methylation-, and development-related genes in cloned mouse blastocysts. *Cloning and Stem Cells*.

[277] Wee G, Shim JJ, Koo DB, Chae JI, Lee KK, Han YM (2007). Epigenetic alteration of the donor cells does not recapitulate the reprogramming of DNA methylation in cloned embryos. *Reproduction*.

[278] Martinez-Diaz MA, Che L, Albornoz M (2010). Pre- and postimplantation development of swine-cloned embryos derived from fibroblasts and bone marrow cells after inhibition of histone deacetylases. *Cellular Reprogramming*.

